# Circulating Irisin and esRAGE as Early Biomarkers of Decline of Metabolic Health

**DOI:** 10.3390/jcm9020454

**Published:** 2020-02-06

**Authors:** Elena Dozio, Elena Vianello, Clementina Sitzia, Federico Ambrogi, Stefano Benedini, Silvia Gorini, Benedetta Rampoldi, Roberta Rigolini, Lorenza Tacchini, Massimiliano Marco Corsi Romanelli

**Affiliations:** 1 Department of Biomedical Sciences for Health, Università degli Studi di Milano, Via Mangiagalli 31, 20133 Milan, Italy; elena.vianello@unimi.it (E.V.); stefano.benedini@unimi.it (S.B.); lorenza.tacchini@unimi.it (L.T.); mmcorsi@unimi.it (M.M.C.R.); 2 Residency Program in Clinical Pathology and Clinical Biochemistry, Università degli Studi di Milano, 20133 Milan, Italy; clementina.sitzia@unimi.it; 3 Department of Clinical Sciences and Community Health, Laboratory of Medical Statistics, Biometry and Epidemiology “G.A. Maccaro”, Università degli Studi di Milano, Via Vanzetti 5, 20133 Milan, Italy; federico.ambrogi@unimi.it; 4 Instrumentation Laboratory—A Werfen Company, R&D Department, Viale Monza 338, 20128 Milan, Italy; sgorini@werfen.com; 5 Service of Laboratory Medicine1-Clinical Pathology, IRCCS Policlinico San Donato, Piazza E. Malan, San Donato Milanese, 20097 Milan, Italy; benedetta.rampoldi@grupposandonato.it (B.R.); roberta.rigolini@grupposandonato.it (R.R.)

**Keywords:** *AGE*, cardiometabolic risk, cRAGE, esRAGE, irisin, sRAGE

## Abstract

A decline in metabolic health may take place before observing any alteration in the levels of the traditional metabolic markers. New indicators of metabolic derangement are therefore compelling. Irisin is a myokine with important metabolic functions. The role of irisin as a metabolic biomarker in humans has not been fully established yet. We quantified plasma irisin and esRAGE in 106 apparently healthy individuals and we performed a cluster analysis to evaluate their associations with metabolic profile. Plasma levels of various traditional markers of metabolic risk (i.e., glucose and lipid levels) were all within the ranges of normality. We identified two clusters of individuals. Compared to cluster 2, individuals in cluster 1 had higher irisin levels, a metabolic profile shifted toward the limits of the reference ranges and lower esRAGE levels. The traditional metabolic blood tests seem not to be enough to identify a metabolic decline early. Irisin increase and esRAGE decrease may reflect a metabolic derangement at the beginning of its development. The role of these molecules as early biomarkers of decline of metabolic health seems an interesting topic to be further explored.

## 1. Introduction

Irisin is a myokine mainly produced by the skeletal muscle after exercise and exposure to cold through the stimulation of peroxisome proliferator-activated receptor gamma coactivator 1-alpha [1]. In adipose tissue, irisin may promote thermogenesis and energy expenditure by increasing the expression of uncoupling protein 1 and browning of white adipose cells [2]. Irisin has also been shown to have important metabolic functions. In particular, it can decrease glucose level, improve insulin resistance [3,4,5,6,7] and, interestingly, counteract some detrimental effects induced by advanced glycation end products (AGE) [8]. By binding to RAGE (membrane receptor for AGE), AGE may in fact promote inflammation, oxidative stress and endothelial dysfunction [9,10]. AGE formation is the result of normal metabolism, but their production and accumulation are enhanced under inflammation and oxidative stress, two conditions that characterize metabolic derangement.

The soluble receptor for AGE (sRAGE) is recognized as the main protective molecule against AGE. sRAGE is a pool composed by cRAGE, derived by the proteolytic cleavage of the membrane-bound RAGE, and esRAGE, the endogenous secretory form. While esRAGE is the real physiological decoy receptor which protects against AGEs [11,12,13], cRAGE is mainly considered a surrogated marker of inflammation. When AGE bind to RAGE, they promote inflammation, increase RAGE expression and up-regulate the levels of inflammatory enzymes, like metalloproteases (MMP), that, by cleaving RAGE, increase cRAGE level [9,14,15,16,17]. Notably, when AGE increase and reach very high levels, such as in diabetes mellitus (DM), AGE positively correlate with esRAGE too, maybe as a potential counter-regulatory mechanism to protect against AGE-related detrimental effects. Even oral AGE intake may increase serum esRAGE level [18]. Up until now, the optimal irisin level in human has not been established, and controversial results about its levels in different pathological conditions have been observed. In fact, both decreased and increased irisin levels have been observed in DM, insulin resistance and metabolic syndrome [19,20,21,22,23]. If, from one side, irisin reduction might lead to some metabolic changes involved in the onset and progression of a disease, on the other side its up-regulation could be a consequence of an “irisin resistance” state and therefore an attempt to maximize the anti-obesity, anti-hyperglycemic and healthy effects of the molecule.

Considering that a decline in metabolic health may take place before observing any alteration in the levels of the traditional metabolic markers, new indicators of metabolic derangement are therefore compelling. Irisin has important metabolic functions, but its role as a metabolic biomarker in human has not been fully established yet.

To this end, in this study we performed a cluster analysis in apparently healthy individuals to evaluate irisin association with the classical anthropometric and metabolic parameters usually used in the clinical setting for risk stratification, as well as with other emerging indicators of metabolic stress and inflammation, such as total sRAGE and its different forms, esRAGE and cRAGE.

## 2. Experimental Section

### 2.1. Source Population

One hundred and six voluntary subjects were recruited at the IRCCS Policlinico San Donato between October 2017 and April 2018. The inclusion criteria were age >18 years and signed written informed consent. Individuals who met the following criteria were not eligible for the study: body mass index (BMI) <18.5, chronic illnesses (cardiovascular diseases, hematological and rheumatic diseases, inflammatory bowel diseases, chronic renal failure, hypercortisolism, DM, hyper- or hypo-thyroidism, hypertension), history of cancer, alcohol and drug abuse, pregnancy, use of pharmacological therapy, and hospitalization in the previous 2 months. Demographic, clinical and biochemical data were recorded for each individual and are described in detail in the following sections. The study was approved by the Institutional Review Board (Comitato Etico OSR, protocol number 88/int/2016) and all participants gave their written informed consent before enrollment in the study. All procedures were conducted in accordance with the Declaration of Helsinki, as revised in 2013.

### 2.2. Blood Collection and Biochemical Parameters

Blood samples were collected after an overnight fasting. Biochemical parameters (glucose, insulin, triglycerides, total and HDL-cholesterol, uric acid, creatinine, glycated hemoglobin (HbA1c), 25-hydroxy vitamin D (25OHD) and non-esterified free fatty acids (NEFA)) were assayed using Cobas 6000 analyzer (Roche Diagnostics, Milan, Italy) as previously reported [5,24,25]. LDL-cholesterol was calculated with the Friedewald’s formula. For non-routine analyses, plasma-EDTA was separated after centrifugation at 1500× *g* for 15 min and stored at −80 °C until analysis. The homeostasis model assessment of insulin resistance (HOMA-IR) index was calculated as follows: HOMA-IR = fasting insulin (mU/L) fasting glucose (mmoL/L) / 22.5 [26]. A HOMA-IR ≥2.5 suggested insulin resistance. The formula used for the lipid accumulation product (LAP) index was: (waist circumference (WC, cm) − 58) (triglycerides (TG, mmoL/L)) for women and (waist circumference (WC, cm) − 65) ×(triglycerides (TG, mmoL/L)) for men [27].

### 2.3. Anthropometric Measures

Height and weight were recorded to the nearest 0.5 cm and 0.1 kg, respectively, with stadiometers and standard scales. WC was measured using a flexible tape. Body mass index (BMI) was calculated as weight (kg)/height^2^ (m^2^), and waist-to-height ratio (WHtR) as WC (cm)/height (cm), respectively. As defined by WHO, patients were classified as normal weight (BMI 18.5–24.9 kg/m^2^), overweight (BMI 25.0–29.9 kg/m^2^) and obese (BMI ≥30.0 kg/m^2^). A WHtR ≥0.5 indicated central obesity [28]. WC was considered a risk factor when greater than 94 cm for men and 80 cm for women [29].

### 2.4. Enzyme-Linked Immunosorbent Assay (ELISA)

Plasma levels of irisin were measured by an irisin/FNDC5 ELISA assay from Phoenix Pharmaceuticals (EK–067–29, CA, USA). The minimum detectable dose was 1.29 ng/mL. The maximum intra- and inter-assay coefficients of variation were <10% and <15%, respectively. Total sRAGE was quantified by a commercial human ELISA kit from R&D Systems (Human RAGE Duo Set ELISA DY1145, Minneapolis, MN, USA) according to the manufacturer’s instructions. esRAGE was measured by the ELISA assay from B-Bridged International (K1009–1, Santa Clara, CA, USA), which uses a monoclonal antibody able to exclusively bind to esRAGE. The intra- and inter-assay coefficient of variation for the esRAGE kits were 6.37% and 4.78%–8.97%, respectively. cRAGE was obtained by subtracting esRAGE concentration from total sRAGE. The cRAGE to esRAGE ratio (cRAGE/esRAGE) was than obtained. This ratio can be used to determine the relationship between these independently produced isoforms and can lend insight into their unique modulation [30,31,32]. The GloMax^®^-Multi Microplate Multimode Reader was used for photometric measurements (Promega, Milan, Italy).

### 2.5. Glycated Albumin Quantification

Glycated albumin (GA, g/L), albumin and the percentage of glycated albumin (GA%) were measured in plasma by the enzymatic QuantILab^®^ Glycated Albumin assay (Instrumentation Laboratory, Milan, Italy) using the ILab650 system (Instrumentation Laboratory). GA% was automatically calculated by the ILab analyzer as GA/albumin ratio corrected by an arithmetic algorithm which aligned the GA% levels to the HPLC reference method [33,34,35]. The minimum detectable concentration of GA was 1.15 g/L. The maximum intra- and inter-assay coefficient of variations were 2.1% and 1.3% for GA and 1.2% and 1.0% for GA%, respectively.

### 2.6. Statistical Analysis

The quantitative variables were expressed as mean with standard deviation (SD) and median with interquartile range. The qualitative variables were summarized as numbers and percentages. The normality of data distribution was assessed by the Kolmogorov–Smirnoff test. The potential univariate association between irisin and other variables was performed with Spearman’s correlation test. Cluster analysis was used to evaluate the association between irisin and the metabolic profile of the individuals. To explore the metabolic profile of the subjects, the following biochemical variables were considered: age, total cholesterol, LDL, HDL, tryglicerides, NEFA, glucose, HbA1c, insulin, GA, uric acid, creatinine, irisin, sRAGE, esRAGE, cRAGE and cRAGE/esRAGE ratio. The Euclidean distance was used for clustering, after having scaled all the variables to have mean zero and variance 1. A hierarchical clustering algorithm was used with Ward distance as implemented in the hclust function in R software (R package version 1.0.6, R Foundation for Statistical Computing, Vienna, Austria). The silhouette index was used for the choice of the optimal number of clusters. To describe the obtained clusters, a comparison between clusters was performed with Wilcoxon rank sum test with continuity correction. The different clusters were also examined according to the following variables: smoking status, alcohol use, WC (greater than 94 for men or greater than 80 for women), WHtR (0.5 cut-off) and HOMA-IR (2.5 cut-off). The odds ratio and 95% confidence interval (CI) were calculated. A *p* value less than 0.05 was considered significant.

## 3. Results

### 3.1. Demographic, Anthropometric and Clinical Characteristics of the Individuals Included in the Study

The main demographic, anthropometric, clinical and biochemical features of the individuals enrolled in the study are presented in Table 1. The mean age was 43.00 ± 11.72 years and the percentage of male gender in the study group was 54.72%. According to BMI, obesity (BMI ≥ 30.0 kg/m^2^) was observed just in 13 subjects (12.26%). WHtR, an index of central obesity, indicated the presence of this condition in half of the subjects. None of the subjects had a diagnosis of DM, history or presence of cardiovascular diseases, hypertension and/or metabolic syndrome. None were under any drug therapy. Hepatic insulin resistance, evaluated by HOMA-IR ≥2.5, was detected in 25 individuals. The average levels of biochemical parameters were within the ranges of normality. In the study group, irisin and GA median levels were 7.89 ng/mL and 13.13%, respectively (Table 1). Total sRAGE, esRAGE and cRAGE median levels were 642.20, 341.10 and 286.70 pg/mL, respectively, and the ratio cRAGE/esRAGE was 0.82 (Table 1).

### 3.2. Correlations of Irisin and esRAGE with Clinical Parameters

The univariate association of plasma irisin and esRAGE with demographic, anthropometric and biochemical parameters was performed using Spearman’s correlation coefficients, and the results are presented in Table 2. Regarding irisin, we found a direct correlation with age (*r* = 0.387, *p* < 0.001), fasting glucose (*r* = 0.200, *p* < 0.05), total cholesterol (*r* = 0.305, *p* = 0.002), LDL cholesterol (*r* = 0.264, *p* = 0.006), triglycerides (*r* = 0.266, *p* = 0.006), LAP index (*r* = 0.200, *p* = 0.045) and GA (*r* = 0.276, *p* = 0.004). Furthermore, we observed an inverse association with sRAGE (*r* = −0.263, *p* = 0.006) and esRAGE (*r* = −0.200, *p* = 0.05), and a direct correlation with GA/sRAGE (*r* = 0.440, *p* < 0.0001), GA/esRAGE (*r* = 0.327, *p* < 0.0001) and GA/cRAGE (*r* = 0.269, *p* < 0.006).

Regarding esRAGE, we found an inverse correlation with BMI (*r* = −0.421, *p* < 0.0001), waist circumference (*r* = −0.368, *p* < 0.0001), WHtR (*r* = −0.435, *p* <0.0001), HbA1c (*r* = −0.218, *p* = 0.025), fasting insulin (*r* = −0.282, *p* = 0.003), triglycerides (*r* = −0.210, *p* = 0.030), uric acid (*r* = −0.253, *p* = 0.009), HOMA-IR (*r* = −0.261, *p* = 0.007), LAP index (*r* = −0.359, *p* = < 0.001), cRAGE (*r* = −0.329, *p* < 0.001), cRAGE/esRAGE (*r* = −0.743, *p* = < 0.001), GA/sRAGE (*r* = −0.496, *p* < 0.0001), GA/esRAGE (*r* = −0.909, *p* < 0.0001) and irisin (*r* = −0.200, *p* = 0.05). Indeed, we observed a direct association with HDL (*r* = 0.207, *p* = 0.033), sRAGE (*r* = 0.614, *p* < 0.0001) and GA/cRAGE (*r* = 0.344, *p* < 0.001).

### 3.3. Multivariate Analysis

**Figure 1:**
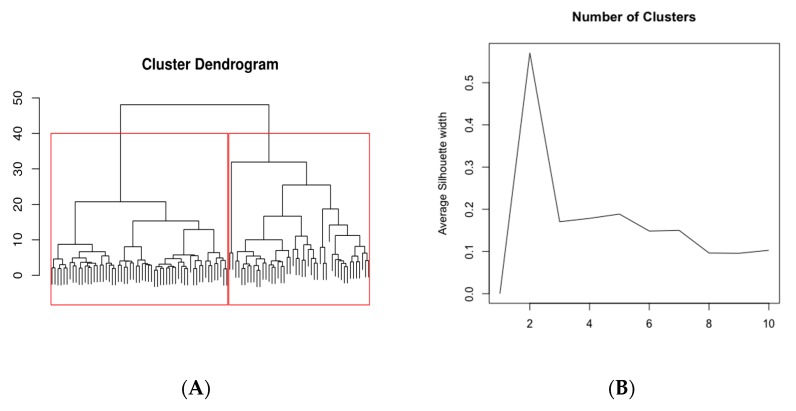
Cluster dendogram and number of clusters. The dendrogram resulting from the application of the hierarchical clustering is reported in the left panel (**A**). Right panel (**B**) shows the number (*n* = 2) of clusters identified according to the silhouette index.

The dendrogram resulting from the application of the hierarchical clustering is reported in Figure 1A. According to the silhouette index (Figure 1B), two clusters of subjects were considered. Cluster 1 includes 47 (44%) subjects, while cluster 2 includes 59 (56%) subjects.

**Figure 2:**
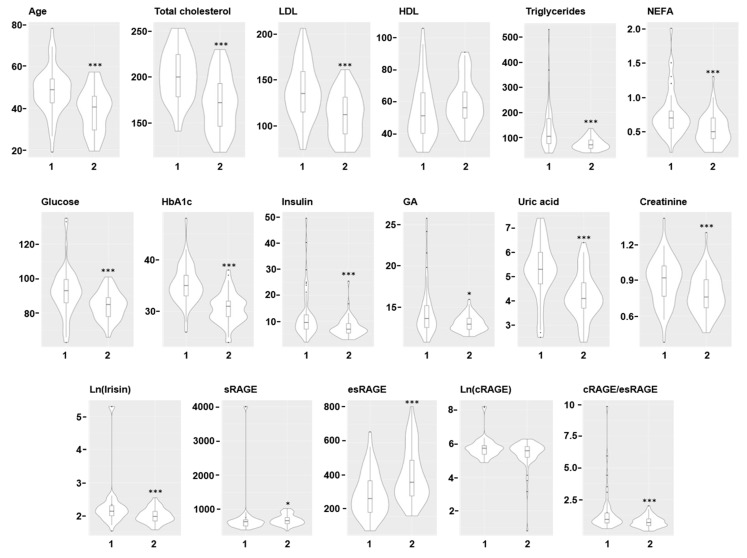
Main characteristics of the individuals included in the two clusters.

The characteristics of the individuals in the two clusters are described in Figure 2 with violin plots. Subjects in cluster 1 are, on average, older than subjects in cluster 2 (*p* < 0.001), have higher mean levels of cholesterol (total and LDL) (*p* < 0.001 for both), triglycerides (*p* < 0.001), NEFA (*p* < 0.001), glucose (*p* < 0.001), HbA1c (*p* < 0.001), insulin (*p* < 0.001), GA (*p* < 0.05), uric acid (*p* < 0.001) and creatinine (*p* < 0.001). Also, irisin, which is shown after a logarithmic transformation, and cRAGE/esRAGE levels are, on average, higher in cluster 1 (*p* < 0.001 for both) (Figure 2). Conversely, the mean sRAGE (*p* < 0.05) and esRAGE (*p* < 0.001) levels are higher in cluster 2. Notably, some individuals with very high irisin, cRAGE and cRAGE/esRAGE levels are clustered in cluster 1. The mean levels (± SD) of the metabolic parameters of individuals in cluster 2 are as follows: fasting glucose, 84.34 ± 7.94 mg/dL; total cholesterol, 161.12 ± 30.20 mg/dL; HDL, 58.92 ± 14.17 mg/dL; LDL, 111.25 ± 24.51 mg/dL; triglycerides, 75.81 ± 23.91 mg/dL; fasting insulin, 7.63 ± 3.68 µU/mL; uric acid, 4.20 ± 0.94 mg/dL; creatinine, 0.79 ± 0.17 mg/dL; NEFA, 0.52 ± 0.23; HbA1c, 30.56 ± 2.94 mmoL/moL.

Subjects’ characteristics in terms of cardio-metabolic risk and risk factors are reported in Table 3. There is a higher percentage of women in cluster 2 (OR 2.71, CI: 1.22–6.03), while the percentage of alcohol consumers and smokers is approximately the same in the two clusters. The percentages of individuals with WC (OR 0.17, CI: 0.07–0.41), WHtR (OR 0.15, CI: 0.06–0.37), and HOMA-IR (OR 0.24, CI: 0.09–0.64) above the cut-offs are higher in cluster 1 than in cluster 2.

The characteristics of the individuals are described with violin plots. Individuals in cluster 1 are, on average, older and have higher mean levels of total cholesterol, LDL-cholesterol, triglycerides, NEFA (non-esterified free fatty acids), glucose, HbA1c (glycated hemoglobin), insulin, GA (glycated albumin), uric acid, creatinine, irisin and cRAGE (membrane-cleaved receptor for advanced glycation end products)/esRAGE (endogenously secreted receptor for advanced glycation end products) levels than subjects in cluster 2. The mean sRAGE (total soluble receptor for advanced glycation end products) and esRAGE levels are higher in cluster 2. *** *p* < 0.0001; * *p* < 0.05.

## 4. Discussion

A decline in metabolic health often precedes the onset of many cardiovascular diseases. Therefore, the early identification of individuals at risk may have important benefits at the social, health and economical levels. Biomarkers provide an easy and minimally invasive means to diagnose, risk stratify, monitor and potentially treat individuals. Despite the availability in the clinical setting of different biomarkers of metabolic homeostasis, mainly related to carbohydrate and lipid metabolism, a decline in metabolic health may take place before observing any alteration in the levels of these traditional biomarkers. Considering that oxidative stress and chronic low-grade inflammation are two early mechanisms that may contribute to the onset and progression of metabolic abnormalities, new indicators of metabolic decline dealing with these early events may be really useful.

In this study, we explored the role of irisin along with the different forms of sRAGE as early biomarkers of metabolic derangement by performing a cluster analysis in a group of apparently healthy individuals. We could identify two clusters of individuals which differed significantly, both in irisin and esRAGE levels. By comparing these two clusters, we could confirm that individuals displaying higher plasma irisin concentration and lower total sRAGE and esRAGE levels had a metabolic profile shifted toward the limits of the reference intervals (higher levels of glucose, HbA1c, total and LDL cholesterol, uric acid, creatinin, insulin, NEFA and lower HDL). The observation that the number of individuals with visceral fat accumulation and insulin resistance was also higher in this group seems to confirm a worsened metabolic profile in this cluster. Some previous studies have already explored the association of irisin with cardiometabolic variables in humans, but they differed from our study for the study population. In fact, they have been performed on children or adolescents [36,37] or adults with metabolic syndrome [38]. To our knowledge, the novelty of our study just deals in the evaluation of irisin and its association with sRAGE and its forms in apparently healthy individuals. The study by Park et al. indicated that irisin increased in individuals displaying metabolic syndrome and cardiovascular risk. Also, studies performed on children and adolescents [36,37] suggested a strong correlation of myokine with unhealthy metabolic parameters and obesity. Our study seems to suggest that irisin up-regulation is a mechanism occurring just at the beginning of a metabolic derangement, maybe as a compensatory mechanism to overcome a potential irisin resistance state. Therefore, irisin might be helpful as an early biomarker of metabolic risk.

Of great interest is also the association between irisin and the different sRAGE forms. AGE are produced continuously in our body. An imbalance between production and detoxification leads to their accumulation and the onset and progression of different disorders, such as cardiometabolic diseases [39]. Although the highest AGE levels may be observed in DM, as a consequence of hyperlycemia, AGE may increase as a consequence of inflammation, redox imbalance, kidney disease and aging [40,41,42]. The damaging effects of AGE may be due both to the direct modification and loss of function of substrates involved in AGE formation (such as matrix proteins, receptors, enzymes, hormones and any type of plasma protein, including albumin) and to RAGE engagement [9,10]. RAGE is usually expressed at low levels in many tissues, but its activation further promotes its expression and the synthesis of reactive oxygen species and pro-inflammatory mediators [9,10]. Together with AGE, RAGE has been associated with the initiation and progression of different disorders, including atherosclerosis, stroke, metabolic syndrome, obesity, DM and kidney diseases [15,43,44,45]. One of the main protective mechanisms against AGE is sRAGE, which is a pool composed of cRAGE, derived by the proteolytic cleavage of the membrane-bound RAGE, and esRAGE, the endogenous secretory form [46]. Considering that esRAGE and cRAGE are produced differently, specific information can be obtained through the quantification of their concentrations. esRAGE is endogenously secreted and its level is usually downregulated by RAGE activation. For this reason, esRAGE is considered the real physiological decoy receptor [11,12,13]. Instead, cRAGE, and the ratio between cRAGE and esRAGE, are mainly considered surrogate markers of inflammation [9,14,15,16,17]. The activation of RAGE promotes inflammation, increases RAGE expression and up-regulates the levels of inflammatory enzymes, like MMP, which by cleaving RAGE increase cRAGE. The final aim is to protect against AGE, both by reducing the availability of membrane RAGE and increasing sRAGE and its protective effects. The observation that the cluster characterized by a worse metabolic status includes individuals with reduced esRAGE and an increased cRAGE/esRAGE ratio and GA, an AGE product, confirmed the existence of a pro-inflammatory background. As one factor in the cRAGE/esRAGE ratio (cRAGE) did not differentially change, we can assume that that the difference in the cRAGE/esRAGE ratio between the two clusters was largely due to changes in the esRAGE level.

To our knowledge, this was the first study that quantified and explored any existing association between irisin, AGE and sRAGE forms in apparently healthy individuals. One previous study was focused on the same molecules but in DM [47].

By considering that irisin may reduce AGE-induced inflammation and endothelial dysfunction via inhibiting ROS-NLRP3 inflammasome signalling [8], the observed positive association between irisin and GA in our individuals reinforces the idea that the up-regulation of irisin also can be a mechanism to counteract the potential detrimental effects induced by AGE, such as inflammation and endothelial dysfunction [8]. Only irisin, but not total sRAGE and esRAGE, was up-regulated in cluster 1. As reported in Table 2, we also evaluated the associations of irisin and esRAGE with GA corrected for sRAGE, esRAGE or cRAGE. Since AGE may drive sRAGE production, these ratios are additional indicators of a poor metabolic profile, and high levels suggest an imbalance between AGE and protective factors. We found a positive association with irisin and a negative correlation with esRAGE. Although both esRAGE and irisin may exert protective effects against AGEs, we observed that at the beginning of a metabolic derangement their levels go in different directions. This study did not explore the molecular mechanisms affecting their circulating levels. It is possible that AGE down-regulate sRAGE expression by activating a pro-inflammatory response through RAGE engagement [9,10] and that irisin is up-regulated as a protective mechanism to improve this situation. On the contrary, the cRAGE/esRAGE ratio had the same trend as irisin. Exploring the trend of these markers during disease progression could help us to better understand their usefulness as early biomarkers of risk and to strengthen the meanings of these preliminary data. Unfortunately, due to the study design and the number of individuals, we could not get information about the diagnostic potential of these markers.

## 5. Conclusions

The traditional metabolic blood tests seem not to be able to identify a metabolic decline early. When blood tests are all within the ranges of normality, people are considered healthy by the practitioners. Our study suggests that the increase in plasma irisin level and the decrease in esRAGE might reflect a metabolic derangement just at the beginning of its development. Although preliminary, our data seem to indicate that their quantification could help physicians to identify a risk and carry out strategies early, such as lifestyle corrections, to reverse it, with important benefits at the social, health and economical levels. However, only longitudinal studies will definitely clarify the role of these molecules as real early biomarkers of metabolic derangement.

## Figures and Tables

**Table 1:** Demographic, anthropometric, clinical and biochemical characteristics of individuals included in the study.

Variable	*n* = 106
Age (years)	43.00 ± 11.72, 43.30 (35.73–51.06)
Male gender (*n*, %)	58, 54.72%
BMI	25.33 ± 4.72, 24.68 (21.66–27.54)
WC (cm)	87.76 ± 15.20, 85.00 (75.00–99.50)
WHtR	0.52 ± 0.08, 0.51 (0.46–0.57)
Fasting glucose (mg/dL)	88.25 ± 12.46, 87.50 (80.75–94.00)
Fasting insulin (microU/mL)	9.71 ± 7.16, 7.52 (5.80–10.96)
HbA1c (mmoL/moL)	32.61 ± 4.04, 32.00 (29.00–35.00)
HOMA-IR	2.24 ± 2.12, 1.62 (1.14–2.44)
LAP index	35.02 ± 41.22, 19.62 (11.65–35.51)
Total Cholesterol (mg/dL)	185.00 ± 33.04, 181.00 (162.00–212.00)
LDL-Cholesterol (mg/dL)	122.80 ± 30.11, 121.00 (99.00–142.00)
HDL-Cholesterol (mg/dL)	57.01 ± 16.23, 53.50 (43.00–67.00)
Triglycerides (mg/dL)	102.30 ± 70.48, 85.50 (60.00–115.30)
Creatinine (mg/dL)	0.83 ± 1.19, 0.84 (0.69–0.96)
GFR (ml/min/1.73 m^2^)	84.09 ± 8.70, 89.00 (80.75–90.00)
Uric acid (mg/dL)	4.68 ± 1.18, 4.70 (3.80–5.43)
NEFA (mg/dL)	0.62 ± 0.30, 0.60 (0.40–0.70)
Obesity (*n*, %)	13, 12.26%
Central obesity (*n*, %)	53, 50.00%
Smoking (*n*, %)	24, 22.64%
Irisin (ng/mL)	13.44 ± 31.99, 7.89 (6.54–7.63)
sRAGE (pg/mL)	720.00 ± 478.60, 642.20 (554.00–746.70)
esRAGE (pg/mL)	377.00 ± 143.20, 341.10 (271.70–462.80)
cRAGE (pg/mL)	343.10 ± 458.10, 286.70 (202.60–343.30)
cRAGE/esRAGE	1.06 ± 1.20,0.82 (0.49–1.22)
GA (%)	13.68 ± 2.34, 13.13 (12.38–14.09)

The table shows the main characteristics of the subjects included in the study. Data are expressed as mean ± standard deviation, median (25th–75th percentiles) or number and proportions. BMI, body mass index; cRAGE, membrane-cleaved receptor for advanced glycation end products; esRAGE, endogenous secretory receptor for advanced glycation end products; GA, glycated albumin; GFR, glomerular filtration rate; HbA1c, glycated hemoglobin; HOMA-IR, homeostatic model assessment of insulin resistance; LAP, lipid accumulation product; NEFA, non-esterified fatty acids; sRAGE, soluble receptor for advanced glycation end products; WC, waist circumference; WHtR, waist-to-height ratio.

**Table 2:** Univariate association of plasma irisin and esRAGE levels with demographic, anthropometric and biochemical parameters of individuals included in the study.

	IRISIN		esRAGE	
	*r*	*p*-Value	*r*	*p*-Value
Age	0.387	**<0.0001**	−0.146	0.1358
BMI	0.141	0.149	−0.421	**<0.0001**
Waist	0.131	0.184	−0.368	**<0.0001**
WHtR	0.136	0.169	−0.435	**<0.0001**
WHR				
Fasting glucose	0.200	**0.050**	−0.165	0.092
HbA1c	0.115	0.239	−0.218	**0.025**
Fasting insulin	0.018	0.855	−0.282	0.003
Total cholesterol	0.305	**0.002**	−0.100	0.310
HDL cholesterol	−0.169	0.083	0.207	**0.033**
LDL cholesterol	0.264	**0.006**	−0.093	0.343
Triglycerides	0.266	**0.006**	−0.210	**0.030**
Uric Acid	0.153	0.117	−0.253	**0.009**
Creatinine	0.037	0.708	−0.038	0.699
GFR	−0.003	0.978	−0.059	0.551
NEFA	0.075	0.442	−0.177	0.069
HOMA-IR	0.060	0.540	−0.261	**0.007**
LAP index	0.200	**0.045**	−0.359	**<0.001**
sRAGE	−0.263	**0.006**	0.614	**<0.0001**
esRAGE	−0.200	**0.050**	-	-
cRAGE	−0.119	0.224	−0.329	**<0.001**
cRAGE/esRAGE	0.005	0.961	−0.743	**<0.001**
GA	0.276	**0.004**	0.123	0.210
GA/sRAGE	0.440	**<0.0001**	−0.496	**<0.0001**
GA/esRAGE	0.327	**<0.0001**	−0.909	**<0.0001**
GA/cRAGE	0.269	**0.006**	0.344	**<0.001**
irisin	-	**-**	-0.200	**0.050**

Associations between variables were explored using Spearman’s correlation coefficients. BMI, body mass index; cRAGE, membrane-cleaved receptor for advanced glycation end products; esRAGE, endogenous secretory receptor for advanced glycation end products; GA, glycated albumin; GFR, glomerular filtration rate; HbA1c, glycated hemoglobin; HOMA-IR, homeostatic model assessment of insulin resistance; LAP, lipid accumulation product; NEFA, non-esterified fatty acids; sRAGE, soluble receptor for advanced glycation end products; WC, waist circumference; WHtR, waist-to-height ratio.

**Table 3:** Subjects characteristics and risk factors in the two clusters of subjects.

Variable	Cluster 1	Cluster 2	OR (95% CI)
Females	15/47 (0.32)	33/59 (0.56)	**2.71 (1.22–6.03)**
Alcohol consumption			
*Moderate*	12/47 (0.26)	19/59 (0.32)	1.11 (0.44–2.87)
*Yes*	15/47 (0.32)	13/59 (0.22)	0.61 (0.23–1.57)
Smoking	10/47 (0.21)	14/59 (0.24)	1.15 (0.46–2.89)
WC ≥ 94 cm (male), ≥ 80 cm (female)	33/45 (0.73)	19/59 (0.32)	**0.17 (0.07–0.41)**
WHtR ≥ 0.5	36/45 (0.80)	22/59 (0.37)	**0.15 (0.06–0.37)**
HOMA-IR ≥ 2.5	17/47 (0.36)	7/59 (0.12)	**0.24 (0.09–0.64)**

CI, confidence interval; HOMA-IR, homeostatic model assessment of insulin resistance; OR, odds ratio comparing cluster 2 with cluster 1; WC, waist circumference; WHtR, waist-to-height ratio.

## References

[1] Brenmoehl J., Albrecht E., Komolka K., Schering L., Langhammer M., Hoeflich A., Maak S. (2014). Irisin Is Elevated in Skeletal Muscle and Serum of Mice Immediately after Acute Exercise. Int. J. Boil. Sci..

[2] Hofmann T., Elbelt U., Stengel A. (2014). Irisin as a muscle-derived hormone stimulating thermogenesis—A critical update. Peptides.

[3] Liu J. (2015). Irisin as an exercise-stimulated hormone binding crosstalk between organs. Eur. Rev. Med. Pharmacol. Sci..

[4] Xin C., Liu J., Zhang J., Zhu D., Wang H., Xiong L., Lee Y., Ye J., Lian K., Xu C. (2016). Irisin improves fatty acid oxidation and glucose utilization in type 2 diabetes by regulating the AMPK signaling pathway. Int. J. Obes. (Lond.).

[5] Benedini S., Dozio E., Invernizzi P.L., Vianello E., Banfi G., Terruzzi I., Luzi L., Romanelli M.M.C. (2017). Irisin: A Potential Link between Physical Exercise and Metabolism—An Observational Study in Differently Trained Subjects, from Elite Athletes to Sedentary People. J. Diabetes Res..

[6] Ye L., Xu M., Hu M., Zhang H., Tan X., Li Q., Shen B., Huang J. (2018). TRPV4 is involved in irisin-induced endothelium-dependent vasodilation. Biochem. Biophys. Res. Commun..

[7] Zhang D., Xie T., Leung P.S. (2018). Irisin Ameliorates Glucolipotoxicity-Associated beta-Cell Dysfunction and Apoptosis via AMPK Signaling and Anti-Inflammatory Actions. Cell Physiol. Biochem..

[8] Deng X., Huang W., Peng J., Zhu T.T., Sun X.L., Zhou X.Y., Yang H., Xiong J.F., He H.Q., Xu Y.H. (2018). Irisin Alleviates Advanced Glycation End Products-Induced Inflammation and Endothelial Dysfunction via Inhibiting ROS-NLRP3 Inflammasome Signaling. Inflammation.

[9] Ramasamy R., Yan S.F., Herold K., Clynes R., Schmidt A.M. (2008). Receptor for advanced glycation end products: Fundamental roles in the inflammatory response: Winding the way to the pathogenesis of endothelial dysfunction and atherosclerosis. Ann. N. Y. Acad. Sci..

[10] Yan S.F., Ramasamy R., Schmidt A.M. (2009). The receptor for advanced glycation endproducts (RAGE) and cardiovascular disease. Expert Rev. Mol. Med..

[11] Choi K., Yoo H., Kim H., Lee K., Seo J., Kim S., Kim N., Choi D., Baik S. (2009). Association between endogenous secretory RAGE, inflammatory markers and arterial stiffness. Int. J. Cardiol..

[12] Di Pino A., Urbano F., Zagami R.M., Filippello A., Di Mauro S., Piro S., Purrello F., Rabuazzo A.M. (2016). Low Endogenous Secretory Receptor for Advanced Glycation End-Products Levels Are Associated With Inflammation and Carotid Atherosclerosis in Prediabetes. J. Clin. Endocrinol. Metab..

[13] Du R., Zhang R.Y., Lu L., Shen Y., Pu L.J., Zhu Z.B., Zhang Q., Hu J., Yang Z.K., Ding F.H. (2018). Increased glycated albumin and decreased esRAGE levels in serum are related to negative coronary artery remodeling in patients with type 2 diabetes: An Intravascular ultrasound study. Cardiovasc. Diabetol..

[14] Raucci A., Cugusi S., Antonelli A., Barabino S.M., Monti L., Bierhaus A., Reiss K., Saftig P., Bianchi M.E. (2008). A soluble form of the receptor for advanced glycation endproducts (RAGE) is produced by proteolytic cleavage of the membrane-bound form by the sheddase a disintegrin and metalloprotease 10 (ADAM10). FASEB J..

[15] Ueno H., Koyama H., Shoji T., Monden M., Fukumoto S., Tanaka S., Otsuka Y., Mima Y., Morioka T., Mori K. (2011). Receptor for advanced glycation end-products (RAGE) regulation of adiposity and adiponectin is associated with atherogenesis in apoE-deficient mouse. Atherosclerosis.

[16] Zhao D., Wang Y., Xu Y. (2012). Decreased serum endogenous secretory receptor for advanced glycation endproducts and increased cleaved receptor for advanced glycation endproducts levels in patients with atrial fibrillation. Int. J. Cardiol..

[17] Monden M., Koyama H., Otsuka Y., Morioka T., Mori K., Shoji T., Mima Y., Motoyama K., Fukumoto S., Shioi A. (2013). Receptor for advanced glycation end products regulates adipocyte hypertrophy and insulin sensitivity in mice: Involvement of Toll-like receptor 2. Diabetes.

[18] Miura J., Yamamoto Y., Osawa M., Watanabe T., Yonekura H., Uchigata Y., Yamamoto H., Iwamoto Y. (2007). Endogenous Secretory Receptor for Advanced Glycation Endproducts Levels Are Correlated With Serum Pentosidine and CML in Patients With Type 1 Diabetes. Arter. Thromb. Vasc. Boil..

[19] Choi Y.-K., Kim M.-K., Bae K.H., Seo H.-A., Jeong J.-Y., Lee W.-K., Kim J.-G., Lee I.-K., Park K.-G. (2013). Serum irisin levels in new-onset type 2 diabetes. Diabetes Res. Clin. Pr..

[20] Liu J.-J., Wong M.D., Toy W.C., Tan C.S., Liu S., Ng X.W., Tavintharan S., Sum C.F., Lim S.C. (2013). Lower circulating irisin is associated with type 2 diabetes mellitus. J. Diabetes Complicat..

[21] Rana K.S., Pararasa C., Afzal I., Nagel D.A., Hill E.J., Bailey C.J., Griffiths H.R., Kyrou I., Randeva H.S., Bellary S. (2017). Plasma irisin is elevated in type 2 diabetes and is associated with increased E-selectin levels. Cardiovasc. Diabetol..

[22] Elizondo-Montemayor L., Mendoza-Lara G., Gutierrez-DelBosque G., Peschard-Franco M., Nieblas B., Garcia-Rivas G. (2018). Relationship of Circulating Irisin with Body Composition, Physical Activity, and Cardiovascular and Metabolic Disorders in the Pediatric Population. Int. J. Mol. Sci..

[23] Saber G.Y., Kasabri V., Saleh M.I., Suyagh M., Halaseh L., Jaber R., Abu-Hassan H., Alalawi S. (2019). Increased irisin versus reduced fibroblast growth factor1 (FGF1) in relation to adiposity, atherogenicity and hematological indices in metabolic syndrome patients with and without prediabetes. Horm. Mol. Boil. Clin. Investig..

[24] Malavazos A.E., Corsi M.M., Ermetici F., Coman C., Sardanelli F., Rossi A., Morricone L., Ambrosi B. (2007). Proinflammatory cytokines and cardiac abnormalities in uncomplicated obesity: Relationship with abdominal fat deposition. Nutr. Metab. Cardiovasc. Dis..

[25] Dozio E., Dogliotti G., Malavazos A., Bandera F., Cassetti G., Vianello E., Zelaschi R., Barassi A., Pellissero G., Solimene U. (2012). IL-18 level in patients undergoing coronary artery bypass grafting surgery or valve replacement: Which link with epicardial fat depot?. Int. J. Immunopathol. Pharmacol..

[26] Matthews D.R., Hosker J.P., Rudenski A.S., A Naylor B., Treacher D.F., Turner R.C. (1985). Homeostasis model assessment: Insulin resistance and beta-cell function from fasting plasma glucose and insulin concentrations in man. Diabetologia.

[27] Kahn H.S. (2005). The “lipid accumulation product” performs better than the body mass index for recognizing cardiovascular risk: A population-based comparison. BMC Cardiovasc. Disord..

[28] Browning L.M., Hsieh S.D., Ashwell M. (2010). A systematic review of waist-to-height ratio as a screening tool for the prediction of cardiovascular disease and diabetes: 0·5 could be a suitable global boundary value. Nutr. Res. Rev..

[29] Alberti K., Zimmet P., Shaw J. (2006). Metabolic Syndrome--A New World-Wide Definition. A Consensus Statement from the International Diabetes Federation. Diabet. Met..

[30] Yamamoto Y., Miura J., Sakurai S., Watanabe T., Yonekura H., Tamei H., Matsuki H., Obata K.-I., Uchigata Y., Iwamoto Y. (2007). Assaying soluble forms of receptor for advanced glycation end products. Arter. Thromb. Vasc. Biol..

[31] Tang S.C., Yeh S.J., Tsai L.K., Hu C.J., Lien L.M., Peng G.S., Yang W.S., Chiou H.Y., Jeng J.S. (2016). Cleaved but not endogenous secretory RAGE is associated with outcome in acute ischemic stroke. Neurology.

[32] Miranda E.R., Fuller K.N., Perkins R.K., Kroeger C.M., Trepanowski J.F., Varady K.A., Haus J.M. (2018). Endogenous secretory RAGE increases with improvements in body composition and is associated with markers of adipocyte health. Nutr. Metab. Cardiovasc. Dis..

[33] Kouzuma T., Usami T., Yamakoshi M., Takahashi M., Imamura S. (2002). An enzymatic method for the measurement of glycated albumin in biological samples. Clin. Chim. Acta.

[34] Kouzuma T., Uemastu Y., Usami T., Imamura S. (2004). Study of glycated amino acid elimination reaction for an improved enzymatic glycated albumin measurement method. Clin. Chim. Acta.

[35] Kohzuma T., Yamamoto T., Uematsu Y., Shihabi Z.K., Freedman B.I. (2011). Basic performance of an enzymatic method for glycated albumin and reference range determination. J. Diabetes Sci. Technol..

[36] Jang H.B., Kim H.-J., Kang J.H., Park S.I., Park K.H., Lee H.-J. (2017). Association of circulating irisin levels with metabolic and metabolite profiles of Korean adolescents. Metabolism.

[37] Nigro E., Scudiero O., Monaco M.L., Polito R., Schettino P., Grandone A., Perrone L., Del Giudice E.M., Daniele A. (2017). Adiponectin profile and Irisin expression in Italian obese children: Association with insulin-resistance. Cytokine.

[38] Park K.H., Zaichenko L., Brinkoetter M., Thakkar B., Sahin-Efe A., Joung K.E., Tsoukas M.A., Geladari E.V., Huh J.Y., Dincer F. (2013). Circulating irisin in relation to insulin resistance and the metabolic syndrome. J. Clin. Endocrinol. Metab..

[39] Chaudhuri J., Bains Y., Guha S., Kahn A., Hall D., Bose N., Gugliucci A., Kapahi P. (2018). The Role of Advanced Glycation End Products in Aging and Metabolic Diseases: Bridging Association and Causality. Cell Metab..

[40] Arsov S., Graaff R., Van Oeveren W., Stegmayr B., Sikole A., Rakhorst G., Smit A.J. (2014). Advanced glycation end-products and skin autofluorescence in end-stage renal disease: A review. Clin. Chem. Lab. Med..

[41] Karumanchi D.K., Karunaratne N., Lurio L., Dillon J.P., Gaillard E.R. (2015). Non-enzymatic glycation of alpha-crystallin as an in vitro model for aging, diabetes and degenerative diseases. Amino Acids.

[42] Limongi D., Baldelli S. (2016). Redox Imbalance and Viral Infections in Neurodegenerative Diseases. Oxidative Med. Cell. Longev..

[43] Basta G. (2008). Receptor for advanced glycation endproducts and atherosclerosis: From basic mechanisms to clinical implications. Atherosclerosis..

[44] Dozio E., Vianello E., Briganti S., Lamont J., Tacchini L., Schmitz G., Corsi Romanelli M.M. (2016). Expression of the Receptor for Advanced Glycation End Products in Epicardial Fat: Link with Tissue Thickness and Local Insulin Resistance in Coronary Artery Disease. J. Diabetes Res..

[45] Dozio E., Vianello E., Bandera F., Longhi E., Brizzola S., Nebuloni M., Romanelli M.M.C. (2019). Soluble Receptor for Advanced Glycation End Products: A Protective Molecule against Intramyocardial Lipid Accumulation in Obese Zucker Rats?. Mediat. Inflamm..

[46] Vazzana N., Santilli F., Cuccurullo C., Davì G. (2009). Soluble forms of RAGE in internal medicine. Intern. Emerg. Med..

[47] Li Z., Wang G., Zhu Y.-J., Li C.-G., Tang Y.-Z., Jiang Z.-H., Yang M., Ni C.-L., Chen L.-M., Niu W.-Y. (2017). The relationship between circulating irisin levels and tissues AGE accumulation in type 2 diabetes patients. Biosci. Rep..

